# HSP70 from the Antarctic sea urchin *Sterechinus neumayeri*: molecular characterization and expression in response to heat stress

**DOI:** 10.1186/s40659-018-0156-9

**Published:** 2018-03-27

**Authors:** Marcelo González-Aravena, Camila Calfio, Luis Mercado, Byron Morales-Lange, Jorn Bethke, Julien De Lorgeril, César A. Cárdenas

**Affiliations:** 1 Laboratorio de Biorrecursos Antárticos, Departamento Científico, Instituto Antártico Chileno, Plaza Muñoz Gamero 1055, Punta Arenas, Chile; 2 Grupo de Marcadores Inmunológicos, Laboratorio de Genética e Inmunología Molecular, Instituto de Biología, Pontificia Universidad Católica de Valparaíso, Avenida Universidad 330, Curauma, Valparaiso, Chile; 3 IFREMER, CNRS, UMR 5244 IHPE « Interactions Hôtes-Pathogènes-Environnements», Université de Montpellier II, Université de Perpignan Via Domitia, Place Eugène Bataillon CC80, 34095 Montpellier Cedex 5, France

**Keywords:** Hsp70, Coelomocytes, Antarctica, Echinoderms, Climate change

## Abstract

**Background:**

Heat stress proteins are implicated in stabilizing and refolding denatured proteins in vertebrates and invertebrates. Members of the Hsp70 gene family comprise the cognate heat shock protein (Hsc70) and inducible heat shock protein (Hsp70). However, the cDNA sequence and the expression of Hsp70 in the Antarctic sea urchin are unknown.

**Methods:**

We amplified and cloned a transcript sequence of 1991 bp from the Antarctic sea urchin *Sterechinus neumayeri*, experimentally exposed to heat stress (5  and 10 °C for 1, 24 and 48 h). RACE-PCR and qPCR were employed to determine Hsp70 gene expression, while western blot and ELISA methods were used to determine protein expression.

**Results:**

The sequence obtained from *S. neumayeri* showed high identity with Hsp70 members. Several Hsp70 family features were identified in the deduced amino acid sequence and they indicate that the isolated Hsp70 is related to the cognate heat shock protein type. The corresponding 70 kDa protein, called *Sn*-Hsp70, was immune detected in the coelomocytes and the digestive tract of *S. neumayeri* using a monospecific polyclonal antibody. We showed that *S. neumayeri* do not respond to acute heat stress by up-regulation of *Sn*-Hsp70 at transcript and protein level. Furthermore, the *Sn*-Hsp70 protein expression was not induced in the digestive tract.

**Conclusions:**

Our results provide the first molecular evidence that *Sn*-Hsp70 is expressed constitutively and is non-induced by heat stress in *S. neumayeri*.

## Background

The success of any organism relies on its niche adaptation and also on its ability to survive to environmental variation (e.g. maintaining homeostasis in stress situations). In this regard, the induction of heat shock proteins (e.g. Hsp70) has been widely reported across different taxa [1]. In the last few decades, the Antarctic Peninsula has shown a strong increase in temperature [2, 3]. Particularly, the increase of shallow seawater temperature could result in a negative impact on various stenothermal species [4]. Sudden changes in sea surface temperature and salinity has been reported in Potter Cove (King George Island) [5] and also in some areas of the Antarctic Peninsula [6]. Recently, high mortality of krill has been reported near to Carlini Station related to significant increases of particulate matter of glacial origin produce by increased temperature [7]. Under this scenario, it has been experimentally determined that Antarctic marine invertebrates may not survive to temperatures between + 5 and + 10 °C. Previous work suggests that fifty percent of the population of clams (*Laternula elliptica*) and limpets (*Nacella concinna*) cannot carry out essential functions between 2 and 3 °C, while individuals of the scallop *Adamussium colbecki* are unable to swim at 2 °C [4, 8]. The thermal stress in *S. neumayeri* is characterized when the sea urchins ceased moving, relax the large spines. This was considered to be the critical thermal maximum in sea urchins [9].

The exposure of an organism to heat stress normally induces a transient expression of heat stress proteins when the protein damage is detected. However, some Antarctic marine species, which have developed in an environment characterized by low temperatures and high stability, have lost the ability to adapt to sudden changes in water temperature [10]. This situation has been demonstrated in two species of Antarctic marine invertebrates (*Odontaster validus* and *Paraceradocus gibber*) which were incapable of up-regulating the expression of Hsp70 at 2 and 6 °C [11]. Nevertheless, it has also been determined that some species of Antarctic mollusks such as *L. elliptica* and *N. concinna*, are able to generate Hsp70 during heat stress at 10 and 15 °C [12]. In *L. elliptica* and *N. concinna* the GRP78 (glucose regulated protein, 78 kDa) is expressed constitutively and in some tissues, could be induced by increased temperature [13].

The sea urchin *Sterechinus neumayeri* is an important member of benthic communities, playing a key role in Antarctic marine ecosystems [14, 15]. In general, sea urchins are considered a good model species for studying the effects of environmental stressors. Sea urchin embryos and coelomocytes have been used as indicators in ecotoxicology studies [16]. In particular, increases in the number of coelomocytes, especially the red cells type, have been documented in many studies reporting different kinds of stress like as injury on skeleton and dermis or environmental contamination by metals, oil soluble fraction and industrial residues [17–20]. Therefore, these cells could be used as stress markers to evaluate the expression of a classical stress protein, such as Hsp70 [16, 17]. Several studies have been conducted on *S. neumayeri* to evaluate environmental stressors including increased temperature, UV-B radiation, salinity, low pH [21–23] and recently, the combined effect of increased temperature and acidification [24–26]. However, none of them have characterized the expression of heat shock proteins in *S. neumayeri*. The impact of acute heat stress in adults of *S. neumayeri* has been evaluated in coelomocytes [27], which are key cells of the innate immune response present in the coelomic fluid. These authors determined an increase in red cell numbers which are involved in antimicrobial activity, changes in the phagocytosis index, modification in the adhesion and spreading capacity of coelomocytes [27]. A recent study showed that several heat shock proteins could be expressed in coelomocytes and digestive gland of *S. neumayeri* [28]. This work also showed that the expression of Hsp70 was not induced in response to water temperatures at 3 and 5 °C. However, no information about gene characteristics and protein expression of Hsp70 from *S. neumayeri* was available.

The aim of this study was to characterize the heat shock protein Hsp70 gene and construct and test a new antibody for protein expression analysis in the coelomocytes of *S. neumayeri*, and to determine whether its expression is modulated after heat stress.

## Methods

### Acute heat stress and sample collection

*Sterechinus neumayeri* individuals were hand collected by SCUBA divers from 10 to 20 m depth in Maxwell Bay, Fildes Peninsula, King George Island (62º12′S–58º57′W), during February 2012. Sea urchins were acclimated in a cold chamber for 1 week at 0.5 °C. They were then transferred directly to the respective aquarium at the selected temperature (5 and 10 °C) and maintained for 48 h. During the heat stress four sea urchins for each condition were recovered at 1, 24 and 48 h and the coelomic fluid was pooled immediately. Three independent experiments were performed with the same experimental conditions.

After heat stress exposure the coelomic fluid was collected into sterile tubes by cutting the peristomial membrane. To avoid aggregation of coelomocytes, the coelomic fluid was collected in the sterile tubes containing the modified Alsever’s solution [29]. Coelomocytes and tissues samples (digestive tract, esophagus, and axial organ) were kept in RNALater (AMBION). After overnight incubation at 4 °C, they were placed at − 20 °C until used.

### RNA extraction and cDNA synthesis

Total RNA was extracted from several tissues (coelomocytes, digestive tract, esophagus and axial organ) using a directly Trizol reagent (Invitrogen, USA) and treated after with DNAse Turbo (Ambion) according to the manufacturer’s instructions. Concentration of total RNA was quantified by measuring absorbance at 260 nm with a spectrophotometer and the integrity of RNA was controlled by agarose gel electrophoresis. First, single-stranded cDNA was synthesized from 1 μg of total RNA prepared with 50 ng/ml oligo-(dT) 12–18 in a 20 μl reaction volume containing 1 mM dNTPs, 1 unit/ml of RnaseOUT and 200 units/ml M-MLV reverse transcriptase in a reverse transcriptase buffer according manufacturer’s instructions (Invitrogen™).

### Cloning and tissue expression of Antarctic sea urchin Hsp70 cDNA

To isolate members of the Hsp70 protein family sequences from the Antarctic sea urchin, primers were designed from a conserved region at the extremities of Hsp70 ORF of invertebrates and vertebrates transcript sequences, Hsp70fw 5′ CCAGCAGTAGGAATTCATCT 3′ and Hsp70rev, 5′ ATCAACCTCCTCGATGGTGG 3′. Coelomocytes cDNA was used as a template for PCR amplification. The PCR program consisted of 3 min at 95 °C, followed by 30 cycles of 95 °C for 1 min, 50 °C for 1 min, 72 °C for 1 min and a final elongation step of 72 °C for 5 min. Amplified products were analyzed on 1.5% agarose gels, cloned in a TOPO vector and sequenced (Macrogen, Korea).

*Sn*-Hsp70 mRNA expression in several tissues was detected by reverse transcription and PCR (RT-PCR). Approximately 1.0 µg of total RNA from different tissues was reverse transcribed as was described above. Amplifications were done using the Hsp70Fw (5′-CCAGCAGTAGGAATTGATC-3′) and Hsp70Rv (5′-AACACCAGCATCCTTCGT-3′), these primers produced an amplicon of 497 pb (Genbank Code: JX035966). The primers were used under the following conditions: 94 °C, 3 min, 50 °C, 1 min, 72 °C, 3 min (1 cycle); 94 °C, 1 min, 60 °C, 1 min, 72 °C, 3 min (30 cycles); 72 °C, 7 min and kept at 4 °C until used. An actin mRNA (GenBank Code: JQ736682) was amplified as a constitutive gene, using the forward (5′-CCTCTCCCTCTACGCATCTG-3′ and reverse 5-AGCGGTGGTTGTGAAAGAGT-3) produced an amplicon of 196 pb.

### Phylogenetic analysis of *Sn*-Hsp70

Phylogenetic analyses were performed using MEGA V.3.0 [30]. The tree was constructed using the Neighbour-Joining method, according to the amino acid sequences alignment using ClustalW. Resultant tree topologies were evaluated by bootstrap analyses based on 1000 re-samplings.

### Quantitative real time PCR during heat stress

The first strand of cDNA was synthesized as describe above and stored at − 20 °C until used for qPCR. Real time PCR was carried out to determine whether acute changes in *Sn*-Hsp70 expression could be detected from coelomocytes sampled at 1, 24 and 48 h during heat stress. The primers qHsp70 Fw: GCAGAGGCATACCTTGGAAA and qHsp70 Rv: AATGGCAGCTGCAGTAGGTT used were determined from Hsp70 cDNA sequence of *S. neumayeri* obtained in this study to produce a PCR product of 152 bp. The reactions were composed of 2.0 μl cDNA diluted at a 1:10 ratio, 0.5 μl of each primer (5 mM) adding 2 μl of Brillant II SYBR Green QPCR master mix (Stratagene) and 2 μl ultra pure water to complete 25 μl final reaction volume. PCR amplification was performed in an Agilent MX3000P Thermocycler, consisted of 40 cycles at 95 °C for 10 min, 95 °C for 15 s, 60 °C for 15 s, and 72 °C for 15 s. A melting curve analysis was done at the end of each PCR reaction to confirm the specific amplifications. For further analysis of the expression level, the crossing points (CP) were determined for each sample using the MxPro™ software (Agilent Technologies). The expressions of the actin, 28S and 18S rRNA were quantified separately and the Excel based application Best-Keeper was used to determine stable housekeeping genes, being gene 28S rRNA the most stable [31]. Specificity of qPCR product was determined by melting curve analysis. The relative expression level of *Sn*-Hsp70 was calculated based on the 2^−DDCP^ method using the 28S ribosomal gene of *S. neumayeri* (Fw GGGTATAGGGGCGAAAGACT, Rv GTCGGGCCTCTTACCAATTT) (GenBank Code: KC218476) as the reference gene [32]. Data was subjected to the ANOVA test and post hoc comparison [33] and the analysis was performed using Graph Pad Prism (GraphPad Software, San Diego California, USA).

### Protein extraction

Protein extraction was carried out in treating coelomocytes. Samples were homogenized in a FastPrep homogenizer (MP Biomedicals) with lysis buffer [20 mM Tris, 100 mM NaCl, 0.05% Triton X-100, 5 mM PMSF, 5 mM EDTA, 0.2% inhibitory cocktail protease (SIGMA)]. The homogenate was centrifuged at 12,000*g*, for 20 min at 4 °C, and the supernatant was rescued and stored at − 80 °C until use.

To quantify proteins, the bicinchoninic acid method (BCA kit, Thermo) was carried out. Briefly, each sample was seeded (in duplicate) at a dilution of 1: 5 (25 μl final) in 96 well plates, then the mixture of reagents A and B of the protein quantification kit (1:50) was added. The plate was incubated in darkness for 30 min at 37° C. Finally, the plate was read on a VERSAmax plate reader (Molecular Device) at 562 nm. In addition, a calibration curve was made using bovine albumin serum (BSA) as a standard protein.

### Immune detection of *Sn*-Hsp70

To study *Sn*-Hsp70 at proteomic level, an antibody was produced against a selected epitope sequence obtained from the alignments performed with several Hsp70 proteins corresponding to carboxyl terminal domains (CSEVITWLDANQLAEKDE). The polyclonal monospecific antibody was generated in mouse and purified by affinity chromatography [34]. In this study, we strictly obeyed the animal protocols approved by the Ethics Committee of *Pontificia Universidad Católica de Valparaíso* for Animal Care and Use.

For antibody validation, a western blot was performed with a pool of four samples of coelomocytes lysates from unstressed and thermally stressed sea urchins. 15 μg of total protein of the sample was separated in a 12% SDS/PAGE gel. The gel was then transferred to a nitrocellulose membrane and blocked with BSA 3% for 2 h at 37 °C and washed with PBS-Tween (PBST) 0.2% 3 times for 7 min. Later, the membrane was incubated with the first antibody (immune purified anti-Hsp70) (200 ng/μl) for 90 min at 37 °C. After 3 washes, the second antibody (Goat anti-Mouse IgG (H+L)-HRP), Polyclonal, Thermo) at a 1:7000 dilution was added and incubated at 37 °C for 60 min. The membranes were revealed by chemiluminescence using Pierce ECL Western Blotting Substrate. Images were taken using MF-chemisBIS 20 from Bio Imaging Systems. In addition, in order to demonstrate the presence of Hsp70 during the thermal stress assays, coelomocytes samples (pool of 4 lysates, of each sampling time and thermal stress) were analyzed by western blot.

For indirect ELISA, samples of four different sea urchins coelomocytes and digestive tract lysates (from each sampling time and thermal stress) were seeded (by triplicate) in a 96-well plates (NUNC MaxiSorp). Each well was incubated with 100 µl of sample at 30 ng/µl of total protein for 12 h at 4 °C. Then, the plate was blocked with 1% BSA for 2 h at 37 °C and the immune purified anti-Hsp70 antibody was added at 40 ng/µl for 1.5 h. Subsequently, the second antibody was added at a 1:7000 dilution for 1 h. Afterwards, 100 µl per well of TMB (3,3′, 5,5;-tetramethylbenzidine) chromogen solution was added and incubated for 30 min at room temperature. Reaction was stopped with 50 µl of 1 N sulfuric acid and read at 450 nm on a VERSAmax microplate reader (Molecular Device). Between incubations, the plate was washed three times with PBST 0.05% with a Microplate washer. Mean and SD was calculated with all data. For ANOVA statistical analysis P < 0.05 was used.

A native Hsp70 was detected in coelomocytes by immunofluorescence as follows: coelomocytes were cytocentrifuged on slides coated with poly-lysine (Sigma), permeabilized with 0.1% Triton X-100 and successively incubated overnight with anti-Hsp70 antibody, followed by goat anti-mouse FITC diluted at 1:50 in PBS–Tween 20 (PBST) 0.1% containing 0.005% Evans blue (Sigma Diagnostics) and propidium iodide. The slides were then washed and observed by confocal microscopy Leyca Model DMI 190 3000 [35]. Negative controls were incubated without the primary antibody.

## Results

### Identification of the Hsp70 transcript expressed in *S. neumayeri*

By PCR approach using primers designed from a conserved region of Hsp70 of invertebrates and vertebrates, we obtained (using total cDNA from coelomocytes) a 1991 bp cDNA that codes for an open read frame of 651 amino acids.

**Fig. 1:**
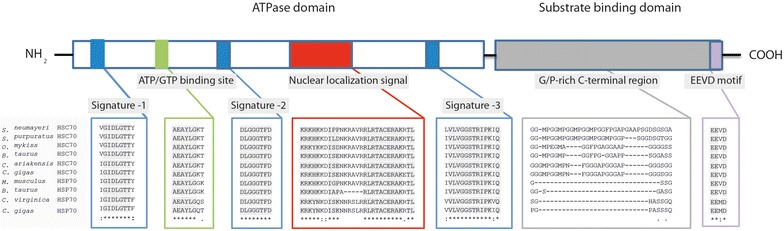
Schematic representation of cognate heat shock protein and multiple alignment of the Sn-Hsp70 with those other cognate and inducible Hsp70 sequences. Blue boxes represent different signatures of Hsp70. Green box represent the ATP/GTP binding site. Red box represent the nuclear localization signal. Grey box represent a multiple tetrapeptide GGMP motif. Purple box represent an EEVD motif. Sequences highlighted in gray indicate conserved residues. Accession Numbers: *Strongylocentrotus purpuratus*: XP 802057; *Oncorhynchus mykiss*: P08108; *Bos taurus*: P19120; *Crassostrea ariakensis*: AAO41703; *Crassostrea gigas*: AAD31042; *Mus musculus*: AAA37859; *Bos Taurus*: NP776769; *Crassostrea virginica*: CAB89802; *Crassostrea gigas*: BAD15286

Similarity analysis of cDNA revealed a close match with other known Hsp70. The nucleotide sequence (GenBank Number JX035966) showed significant sequence homologies with those of other Hsp70, such as the purple sea urchin *Strongylocentrotus purpuratus* and vertebrates. The Hsp70 DNA sequence has the highest degree of identity with *S. purpuratus* (89%). Additionally, the translate sequence protein of *S. neumayeri* showed strongest identity with *S. purpuratus* (88%). On the other hand, comparison of the *S. neumayeri* Hsp70 amino acid sequence with cognate Hsc70 also showed high identity with other organisms such as shrimp, bovine, fish, mice and humans. The partial sequence also contains three Hsp70 signatures: IHLGTTYS, DLGGGTFD and LVLVGGSTRIPKIQ in the N-terminal segment (Fig. 1). The other conserved motifs of Hsp70 family, such as the Nuclear Targeting sequence (KRKHKKDIPPNKRAVRR) and a putative ATP binding site (AEAYLGK) were identified. Four repeated GGMP motifs were also observed, located in the carboxy-terminal domain (Fig. 1). Multiple sequence alignment of *S. neumayeri* Hsp70 with cognate and inducible Hsp70 amino acid sequences revealed that they were highly conserved at the characteristic signature of the Hsp family protein. However, when comparing the N terminal and C terminal domain, the C terminal domain was more variable than the N terminal. The major difference was produced in the highly repeated GGMP motif which is different to the inducible isoform of Hsp70 from vertebrates and invertebrates.

**Fig. 2:**
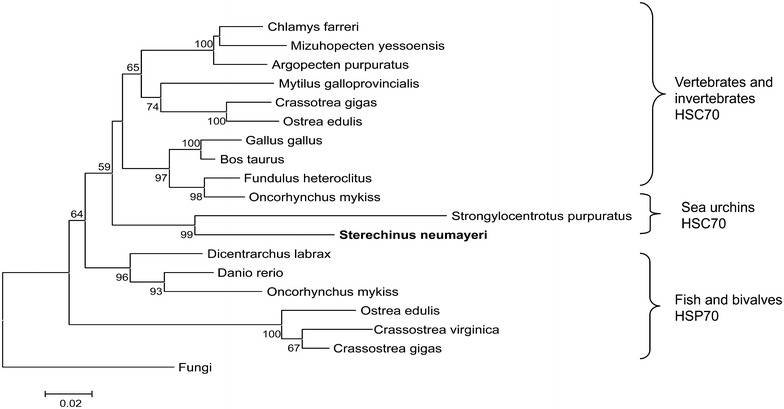
Phylogenetic relationships among Hsp70 family members. The tree was constructed by Neighbour-Joining method. Numbers at each branch indicate the percentage of bootstrap values after 1000 replications. Scale bar represent the amino acid substitutions per site for a unit branch length

A phylogenetic tree of the amino acid sequences of the Antarctic sea urchin *S. neumayeri Sn*-Hsp70 was constructed using a program CLUSTAL and MEGA. The Hsp70 family can be separated into cognate proteins (Hsc70), that are constitutively expressed, and inducible Hsp70 that increase in concentration in the cell after stress conditions. *S. neumayeri* protein sequence was placed together with the predicted sequence of *S. purpuratus* and their branch was connected to characteristic Hsc70 for vertebrate and invertebrates (Fig. 2). On the other hand, the inducible Hsp70 split with another differentiated cluster including fish and mollusk inducible Hsp70. A characteristic feature of Hsc70 is the presence in the carboxy-terminal domain of a binding site that interacts with several cofactors implicated in the regulation of cytosolic and nuclear heat shock cognate proteins.

### Constitutive expression of *Sn*-Hsp70 during heat stress at transcript and protein level

**Fig. 3:**
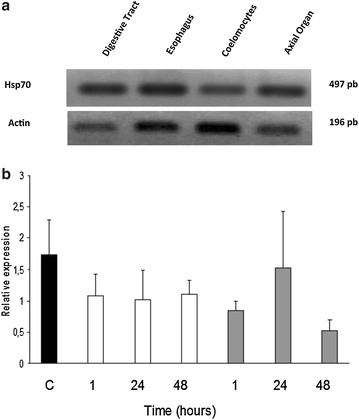
Expression analysis of *Sn*-Hsp70 mRNA. **a** Expression of Hsp70 in tissue by RT-PCR. **b** *Sn*-Hsp70 mRNA expression was measured by qPRC in coelomocytes following heat stress. Results are mean ± SE of three independent experiments carried out on a pool of four sea urchins from control group at 0.5 °C (black) and thermal stressed exposed to 5 °C (white) and 10 °C (grey) during 1, 24 and 48 h. The housekeeping gene used in the qPCR was the 28S rRNA. The primers efficiency was 112 and 109% for 28S rRNA and *Sn*-Hsp70, respectively

Expression of *Sn*-Hsp70 mRNA showed that *S. neumayeri* Hsp70 gene was expressed in all examined tissues (Fig. 3a).

After heat stress, the expression of *Sn*-Hsp70 gene in coelomocytes was not modulated and not different compared to non-stressed animals (Fig. 3b). The ANOVA test of the temperatures (df = 2, F = 2.73, P = 0.75) and time (df = 3, F = 2.6, P = 0.101) do not show any significant effect of heat stress (5 and 10 °C) on gene expression (Fig. 3b).

**Fig. 4:**
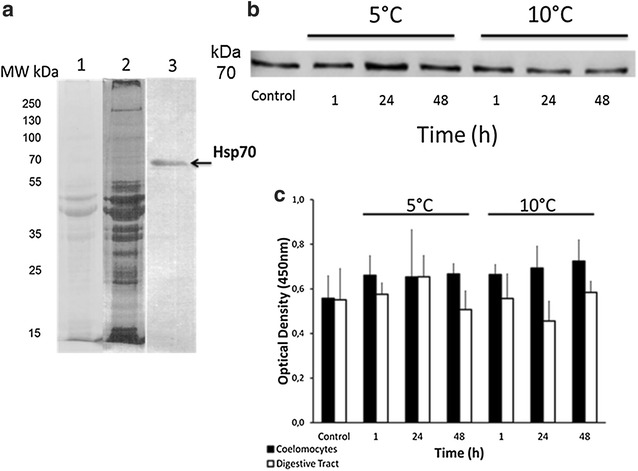
Protein detection of *Sn*-Hsp70 in *S. neumayeri*. **a** Validation of anti-Hsp70. Line 1: SDS-PAGE proteins profile of coelomocytes lysates stained with coomassie blue. Line 2: SDS-PAGE proteins profile stained with silver. Line 3: western blot for antibody validation show only a specific band (arrow) in the expected molecular weight (70 kDa), the antibody not present nonspecific recognition in other molecular weights. **b** Western blot to detect *Sn*-Hsp-70 in coelomocytes during the acute heat stress. **c** Indirect ELISA detection by Optical density to 450 nm of *Sn*-Hsp-70 protein in coelomocytes and digestive tract. Differences in *Sn*-Hsp70 detection are not significant by ELISA (P < 0.05)

In order to confirm this result at the protein level, lysates of total coelomocytes and digestive tract of unstressed and thermally stressed sea urchins were evaluated. Western blot of coelomocytes showed that the secondary antibody detected an apparent band with molecular weight of 70 kDa (Fig. 4a). Additionally, western blot showed some minor intensity changes in some groups of lysates of coelomocytes (Fig. 4b), such as a more intense band at group 5 °C for 24 h that could correspond to an over expression of *Sn*-Hsp70 protein. However, by ELISA the slight changes in Hsp detection in the experimental groups were not significant; indicating that no over expression of *Sn*-Hsp70 protein occurs under the evaluated stress conditions in coelomocytes and digestive tract tissues (Fig. 4c).

**Fig. 5:**
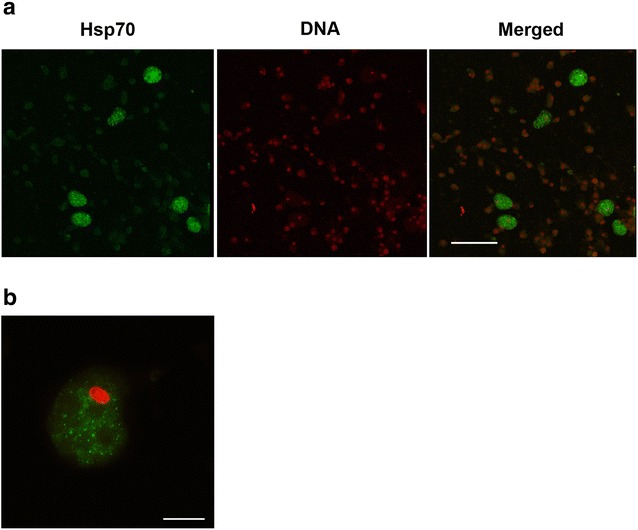
Confocal images of *Sn*-Hsp70 proteins in coelomocytes. **a** Coelomocytes labeled for Hsp70 proteins were expressed in large coelomocytes with an eccentric nucleus. Bars = 50 μm. **b** The positive coelomocytes have intense labeling of the cytoplasm. Bars = 10 μm. Circulating coelomocytes were labeled for DNA (red) and Hsp70 (green)

Coelomocytes of *S. neumayeri* analyzed with light microscopy, showed four conventional morphologies. It was also possible to observe a particular cell morphotype in the coelomic fluid under normal and heat stress conditions. These cells are characterized by their large size in comparison to other coelomocytes. Coelomocytes are oval shaped and their size ranges from 10 to 20 μm, with higher vacuolated cytoplasm and nucleus eccentrically placed. When using confocal imaging, these cells were found to be strongly positive for the anti-Hsp70 antibody when compared to control cells (Fig. 5a). The positive coelomocytes have intense labeling and showed the Hsp70 proteins widely distributed throughout the cytoplasm, however *Sn*-Hsp70 proteins are not present inside the vesicles distributed in the cytoplasm (Fig. 5b).

## Discussion

The identification of a complete DNA sequence of Hsp, named *Sn*-Hsp70, and their detection at the protein level in the coelomocytes of *S. neumayeri* was achieved. *Sn*-Hsp70 sequence includes the typical signatures of heat shock proteins expressed from others organism. The three signatures are well conserved in the *Sn*-Hsp70 (GIDLGTTY, DLGGGTFD, LVLVGGSTRIPKIQ) and these signatures are indicative that this protein belongs to the HSP family [30]. Additionally, the sequence has two other characteristic motifs, the ATP/GTP active site implicated in the ATP hydrolysis [36] and the bi-partite nuclear targeting sequence. These motifs are selective for the translocation of Hsp70 protein into the nucleus [37].

Several Hsp70 in marine invertebrates have approximately two and four repeats of this motif in their carboxy-terminal domain [38]. Several sequences obtained from the Antarctic krill *Euphausia superba* have an identical number of repetitions [39]. The presence of this tetrapeptide in the *Sn*-Hsp70 sequence may indicate that this region could provide binding sites for the chaperone cofactor, but further studies are required to identify the function of this complex in the heat shock protein regulation. Phylogenetic analyses between *Sn*-Hsp70 and other heat shock proteins in vertebrates and invertebrates, showed that *Sn*-Hsp70 had similarities with proteins that belong to the cognate group of heat shock proteins. Based on the conserved motif and phylogenetic analysis, we can conclude that the identified protein is closely related to the Hsc70 subfamily. Additionally, we showed a constitutive expression of *Sn*-Hsp70 in *S. neumayeri* coelomocytes during heat stress at transcript and protein level.

The Hsps are a group of highly conserved proteins, of varying molecular weight (16–100 kDa), produced in vertebrates and invertebrates when they are exposed to cellular stress [40]. The inducible Hsp70 genes are typically activated in response to increased temperatures [41]. Predicted ocean warming scenarios suggest that Antarctic marine stenotherms may be severely affected by increased water temperature, negatively influencing their physiology and biological functions [42]. The classical heat shock response has been demonstrated in the Antarctic mollusks *L. elliptica* and *N. polaris* at 10 and 15 °C for, respectively [12]. Nevertheless, some specificity in Hsp70 expression has been shown in Antarctic organisms, as found in the present study. The Antarctic fish *Trematomus bernacchii*, do not synthesize any size class of Hsp in response to heat shocking or heavy metal stressors [43]. Moreover, the Antarctic sea star *O. validus* could not over express the inducible form after heat stress at 2 or 6 °C, or at elevated temperatures of 10 or 15 °C [7]. Other studies have demonstrated the lack of overexpression in response to thermal stress in stenothermal Antarctic organisms such as the gammarid *P*. *gibber* and the starfish *O. validus* [1]. Results showed that high temperatures did not produce a significant up regulation of Hsp genes in *P. gibber* and *O. validus*, a close related species to sea urchins. These marine invertebrates have lost the capacity to overexpress the Hsp in response to heat stress [11]. This lack of classical heat shock response during the entire acute thermal stress condition could be explained by the absence of positive selection pressure, in cold water species that have lived on a highly stable marine environmental [43]. Unfortunately, only partial nucleotide sequence of *O. validus* and *P. gibber* Hsp70 were available. Therefore, it is difficult to conduct a phylogenetic analysis of this sequence in Antarctic marine invertebrates.

Recently, several Hsp cytoplasmic chaperones from Antarctic krill were characterized using bioinformatics tools. Ten sequences showed similar identity to the classic inducible form of Hsp70 [39]. In contrast, we demonstrate the absence of variation of expression of *Sn*-Hsp70 transcripts during heat stress in coelomocytes of *S. neumayeri*. Due to the PCR approach used in this study, we cannot exclude the presence of other inducible Hsp70 variants in *S. neumayeri*. However, immune detection and ELISA analysis of Hsp70 protein in *S. neumayeri* showed no significant variation at protein level, using a polyclonal antibody that should be able to recognize Hsp70 variants (Hsc70 and Hsc70). This absence of induction of Hsp70 during heat stress detected at protein level could be in agreement with a constitutive expression of Hsp70 in *S. neumayeri*.

The constitutive expression of *Sn*-Hsp70 during heat stress could be a particular response of Antarctic organisms adapted to such stable low temperature environment. Because sea urchins are osmoconformers, any changes in water temperature may influence the coelomic fluid, having a significant influence on *Sn*-Hsp70 expressed in coelomocytes. Furthermore, *Sn*-Hsp70 could play a role in immune response, like in mammalians and, in many cell types, elicits changes in their activity and production of several cytokines and adhesins [44, 45]. Therefore, further studies may analyze the role of Hsp70 of *S. neumayeri* in the context of the immune response at low temperatures.

## Conclusions

Antarctic marine invertebrates have evolve in a stable cold thermal environment. Due to their adaptation to those cold water environments, some Antarctic marine invertebrates have lost the classic heat shock response. The question addressed in this study was whether *S. neumayeri* has a significant up regulation of heat shock protein activity during the acute temperature challenge. Our results provide the first molecular evidence that Sn-Hsp70 is expressed constitutively and is non-induced by heat stress in *S. neumayeri*.
